# Supplementary material for: Diversity and relative abundance of bird species in the two habitat types of Dokima forest Awi zone, Ethiopia
Source: PLoS One. 2023 Mar 21;18(3):e0281595. doi: 10.1371/journal.pone.0281595 (PMC10030032; doi:10.1371/journal.pone.0281595)
Supplement: Attachment — Submitted filename: Response to Reviewers.docx [file pone.0281595.s002.docx]

**Response to the reviewer’s comments:**

**Comment 1:** One-way ANOVA for each, So if differences between habitats is your focus- then do an ANOVA that compares the habitats regardless of season and then show differences between seasons in each habitat separately with a plot (and then you could show your ANOVA results for the seasons separately).  Conversely if its differences between seasons- then show your ANOVA that compares the seasons regardless of habitat and then show differences between habitats in each season separately with a plot (and then you could show your ANOVA results for the habitats separately).

**Answer:** Revised as per the suggestion.

**Comment 2:** Secondly your sorensons index. You give two different equations for it and then after explaining that it ranges from 0 to 1 you give results that are greater than one.

**Answer:** Revised as per the suggestion.

**Comment 3:** We note that Figure 1 in your submission contain [map/satellite] images which may be copyrighted. All PLOS content is published under the Creative Commons Attribution License (CC BY 4.0) or remove the figure

**Answer:** Revised as per the suggestion, the figure is removed from the manuscript.

**Comment 4:** Abstract: Please add one sentence why is this study important (e.g. lack of knowledge on bird ecology in Afrotropical highlands) and specify what are the focal habitat types right from the beginning. Please delete F-statistics, p-values and df.

**Answer:** Revised as per the suggestion.

**Comment 5:** Introduction: There is a lot of redundant information, some of them are even repetitive (e.g. that about woodpecker richness). On the other hand, readers do not know from this text what are the aims of the study. So, I suggest building the Introduction as follows (the points roughly represent intended paragraphs): a) importance of Afrotropical forests for global biodiversity, b) threats to these forests and outcomes of these pressures (some parts are cleared and transformed to shrub land), c) importance of birds in ecological research, d) birds of Ethiopian highland forests + lack of knowledge on their abundance and species richness in different habitat types of this environment, e) study aims.

**Answer:** Revised as per the suggestion.

**Comment 6:** Study area and study site: Please reduce the information on administrative divisions, state simply where the study was conducted and describe briefly the environmental conditions (climate, biotopes) in the area. All the information can be presented in 5-8 sentences. The justification for selecting the study area for research should be moved to the Introduction.

**Answer:** Revised as per the suggestion.

**Comment 7:** Study design and data collection: Please state briefly the sampling period and remove the information about the preliminary survey as it is irrelevant. Description of the habitats should be moved to the previous chapter. Description of bird counts must be elaborated since some sentences are duplicated (but differ in the specific information – see 30m vs. 50m radius!). Many of the information are redundant (e.g. readers do not need to know your bird identification techniques or how did you make your notes on bird detections). When writing this part, please have in mind that it serves for potential reproduction of your research by someone, not for justification of your bird identification skills. Consider the presentation from this perspective.

**Answer:** Revised as per the suggestion.

**Comment 8:** Data analysis: Please describe in more detail the ANOVA. You state that it was used for testing the differences between seasons, but you obviously used it to test for differences between habitats, too. Moreover, the Sorenson similarity index cannot work in the form presented in the text. It is defined as a proportion of species common to two assemblages. So, it cannot be calculated as a number of species common to both habitats divided by the sum of the total numbers of species recorded in respective habitats. If both habitats contained the same 10 species, then it would be 10/(10+10) = 0.5, whereas the correct value of this index should be 1.0. Please revise the formula.

**Answer:** Revised as per the suggestion.

**Comment 9:** Results and Discussion: I do not see any reason for merging these two parts. It makes the text very unclear and reduces the true discussion to minimum. Please separate results to a specific Results section (its organization into subchapters can be the same as it is) and then write a new Discussion section. In Discussion, we need to learn the interpretation of the patterns presented in the Results section, i.e. why there were the differences between the habitats and seasons observed. In addition, please make a comparison with other studies from Afrotropical region in general and from the mountain ecosystems in particular.

**Answer:** Revised as per the suggestion.

**Comment 10:** Conducting the research. Based on the information presented, it seems that you used mainly visual detections. This is quite unusual as 90%+ birds are recorded as aural detections in forest conditions. At the same time, you state that birds were recorded by experts and experiences researchers, so it sounds odd that “binoculars and bird guide books were used” – experts just use their knowledge of bird voices. Moreover, I do not know what was the radius used for bird counts – 30m or 50m? Both figures mean that the radii overlapped in forest habitat because points were separated by 30m distance. Finally, we do not know how the distances were measured – by eye after training or by laser range finder? Taken together, the description of data collection must be much more convincing to judge reliability of the data.

**Answer:** Revised as per the suggestion.

**Comment 11:** Data analysis. I think that one-way ANOVA is an appropriate and powerful tool for addressing the questions you asked, but we do not know what the data points are, and what the explanatory variables are. The other analyses are less convincing. The correlations shown in Fig. 4 make little sense, especially those between population and relative abundance – these variables are identical, they only differ in the way of presentation of the same thing. So, their correlation must be 1.0, as also indicated by the plot. Values of the Sorenson index do not correspond to the theoretical background for this index and are most likely wrong.

**Answer:** Revised as per the suggestion.

**Comment 12:** Comparison with other studies. The data on bird abundance remain scarce in Afrotropical highland conditions, but some studies exist – please consult literature from Albertine rift, Mt. Kenya, Kilimanjaro and our work from the Cameroon Mountains to make a thorough assessment of your findings in the context of other studies.

**Answer:** We tried to revise as per the suggestion, but the exact data you referred to us may not be found.

Other comments were also corrected in addition to the above main ones.
